# Supplementary material for: Resolving the full spectrum of human genome variation using Linked-Reads
Source: Genome Res. 2019 Apr;29(4):635–45. doi: 10.1101/gr.234443.118 (PMC6442396; doi:10.1101/gr.234443.118)
Supplement: Supplemental Material [file supp_gr.234443.118_Supplemental_longranger-2.2.zip › longranger-2.2.tar.bz2!/lr2.2-public/lib/stan/cmdstan/stan/doc/api/html/sort__indices_8hpp_source.html]

Stan: src/stan/math/matrix/sort\_indices.hpp Source File


|  |  |
| --- | --- |
|  | Stan  2.5.0  probability, sampling & optimization |


- Main Page
- Related Pages
- Namespaces
- Classes
- Files

- File List
- File Members

All Classes Namespaces Files Functions Variables Typedefs Enumerations Enumerator Friends Macros Pages

- src
- stan
- math
- matrix

sort\_indices.hpp

Go to the documentation of this file.

1 #ifndef STAN\_\_MATH\_\_MATRIX\_\_SORT\_INDICES\_HPP

2 #define STAN\_\_MATH\_\_MATRIX\_\_SORT\_INDICES\_HPP

3

4

5 #include <vector>

6 #include <algorithm> // std::sort

7 #include <iostream>

8

9 #include <stan/math/matrix/Eigen.hpp>

10 #include <stan/math/matrix/meta/index\_type.hpp>

11

12 namespace stan {

13

14  namespace math {

15

23  namespace {

24  template <bool ascending, typename C>

25  class index\_comparator {

26  const C& xs\_;

27  public:

34  index\_comparator(const C& xs) : xs\_(xs) { }

35

44  bool operator()(int i, int j) const {

45  if (ascending)

46  return xs\_[i-1] < xs\_[j-1];

47  else

48  return xs\_[i-1] > xs\_[j-1];

49  }

50  };

51

52

63  template <bool ascending, typename C>

64  std::vector<int> sort\_indices(const C& xs) {

65  typedef typename index\_type<C>::type idx\_t;

66  idx\_t size = xs.size();

67  std::vector<int> idxs;

68  idxs.resize(size);

69  for (idx\_t i = 0; i < size; ++i)

70  idxs[i] = i + 1;

71  index\_comparator<ascending,C> comparator(xs);

72  std::sort(idxs.begin(), idxs.end(), comparator);

73  return idxs;

74  }

75

76  }

77

85  template <typename C>

86  std::vector<int> sort\_indices\_asc(const C& xs) {

87  return sort\_indices<true>(xs);

88  }

89

97  template <typename C>

98  std::vector<int> sort\_indices\_desc(const C& xs) {

99  return sort\_indices<false>(xs);

100  }

101

102

103  }

104 }

105 #endif

stan::math::sort\_indices\_asc

std::vector< int > sort\_indices\_asc(const C &xs)

Return a sorted copy of the argument container in ascending order.

**Definition:** sort\_indices.hpp:86

index\_type.hpp

Eigen.hpp

stan::math::size

int size(const std::vector< T > &x)

**Definition:** size.hpp:11

stan::math::sort\_indices\_desc

std::vector< int > sort\_indices\_desc(const C &xs)

Return a sorted copy of the argument container in ascending order.

**Definition:** sort\_indices.hpp:98

xs\_

const C & xs\_

**Definition:** sort\_indices.hpp:26


---

[ Stan Home Page ]

*© 2011–2014,
Stan Development Team.*
